# Artificial Intelligence impact on decision-making regarding short-term ADT with prostate radiotherapy: ASTuTE clinical trial interim results

**DOI:** 10.1016/j.ctro.2026.101257

**Published:** 2026-08-10

**Authors:** Eric Wegener, Michael Ng, Mario Guerrieri, Timothy N. Showalter, Jeremy de Leon, Sagar Ramani, Marcus Dreosti, Tee Lim, Bradley Wong, Annie Ho, Michael Chao, Zachary Wilczynski, Peter Chong, Scott McClintock, Darren Foreman, Matthew Brown, Stephen McCombie, Kevin McMillan, Marcus Handmer, Mark Frydenberg, Lih-Ming Wong, Dickon Hayne, John Yaxley, Phillip Stricker, Jarad Martin

**Affiliations:** a GenesisCare Lake Macquarie Private Hospital, 3 Sydney St Lake Macquarie, Gateshead, NSW 2290, Australia; b University of Newcastle, Callaghan, NSW 2308, Australia; c GenesisCare St Vincent's Hospital, 41 Victoria Parade, Fitzroy, VIC 3065, Australia; d Department of Medicine, University of Melbourne, Australia; e Artera Inc., Los Altos, CA, USA; f University of Virginia, Department of Radiation Oncology, Charlottesville, VA, USA; g GenesisCare St Vincent's Hospital, Level A, 438 Victoria Street, Darlinghurst, NSW 2010, Australia; h GenesisCare John Flynn Private Hospital, 42 Inland Drive, Tugun, QLD 4224, Australia; i GenesisCare Kurralta Park, Tennyson Centre, 520 South Road, Kurralta Park, SA 5037, Australia; j Fiona Stanley Hospital, 102-118 Murdoch Drive, Murdoch, WA 6150, Australia; k GenesisCare Buderim, 10 King Street, Buderim, QLD 4556, Australia; l GenesisCare Ringwood Private Hospital, 36 Mount Dandenong Road, Ringwood East, VIC 3135, Australia; m Lake Macquarie Urology, Level 3, Suite 2/20 Smart St, Charlestown, NSW 2290, Australia; n Gold Coast Private Hospital, Level 3, 123 Nerang St, Southport, QLD 4215, Australia; o South Terrace Urology, 326 South Tce, Adelaide, SA 5000, Australia; p St John of God Wexford Medical Centre, 3 Barry Marshall Parade, Murdoch 6150, WA, Australia; q Knox Private Hospital, 262 Mountain Hwy, Wantirna, VIC 3152, Australia; r John Hunter Hospital, Department of Urology, Lookout Road, New Lambton, NSW 2305, Australia; s Australian Urology Associates, Ground Floor, 322 Glenferrie Rd, Malvern, VIC 3144, Australia; t Melbourne Urology Group, Suite 2, 141 Grey Street, East Melbourne, Australia; u Medical School, The University of Western Australia, Perth, Australia; v Wesley Urology Clinic, Suite 42, Level 4, Wesley Medical Centre, 40 Chasely Street, Auchenflower, QLD 4066, Australia; w St Vincent's Hospital Sydney, Darlinghurst, New South Wales, Australia

**Keywords:** Prostate cancer, Androgen deprivation therapy, Artificial Intelligence, Biomarkers, Deep learning, Digital pathology, Radiotherapy

## Abstract

This interim analysis of a multicentre implementation trial (*n* = 200) evaluated a multimodal artificial intelligence biomarker's impact on shared short-term androgen deprivation therapy (ST-ADT) decisions in intermediate-risk prostate cancer (IR-PC). Biomarker testing altered 27.5% (CI: 21.4–34.2%) of final decisions. ST-ADT use significantly decreased: 70.3% (CI: 58.5–80.3%) of patients initially electing ST-ADT changed to ‘NO’, versus a 2.4% (CI: 0.5–6.8%) shift to ‘YES’ (*p* < 0.001). Decision changes were significantly greater in unfavourable versus favourable IR-PC (35% v 4%; p < 0.001). These interim findings demonstrate a significant shift toward de-escalation.

## Highlights

• A multimodal AI biomarker was evaluated in intermediate-risk prostate cancer.
• Testing altered 27.5% of shared decisions regarding short-term ADT use.
• Over 70% of patients initially electing ADT changed their decision to forgo it.
• De-escalation was significantly greater in unfavourable intermediate-risk patients.
• Only 15% of this contemporary cohort were predictive biomarker positive.

## 1 Introduction

Short-term androgen deprivation therapy (ST-ADT) has been shown to improve the efficacy of definitive external beam radiotherapy for men with intermediate-risk prostate cancer (IR-PC) [1]. While it has no defined efficacy role in low-dose-rate brachytherapy, ST-ADT remains widely utilised but is associated with toxicities [2] and clinical parameters alone do not reliably identify those who benefit most. Multimodal artificial intelligence (MMAI) clinicopathological biomarkers have been recently utilised for prognostic and predictive information regarding IR-PC risk of metastases and the potential benefit from ST-ADT. While the prognostic biomarker has been validated [3] and the predictive biomarker has been retrospectively validated on phase III trial cohorts [4], real-world data is needed to assess their effects on shared decision-making regarding ST-ADT use.

## 2 Materials and methods

### 2.1 Trial design and eligibility

This Australian wide multicentre implementation trial and prospective registry collect real world data on the impact of MMAI biomarker tests on treatment decisions for men with IR-PC undergoing curative radiotherapy across 28 centres, for the interim analysis. This study utilises a commercially available MMAI platform (ArteraAI), which extracts features from digitised Haematoxylin and Eosin (H&E)-stained biopsy slides and combines them with standard clinicopathologic data to generate both prognostic and predictive outputs. The trial allows for a wide range of radiotherapy schedules, including Stereotactic Body Radiotherapy (SBRT) and focal boosts. All patients provided written informed consent. The full protocol has been previously published [5] (ACTRN 12623000713695).

### 2.2 Study endpoints and assessments

**Fig. 1:**
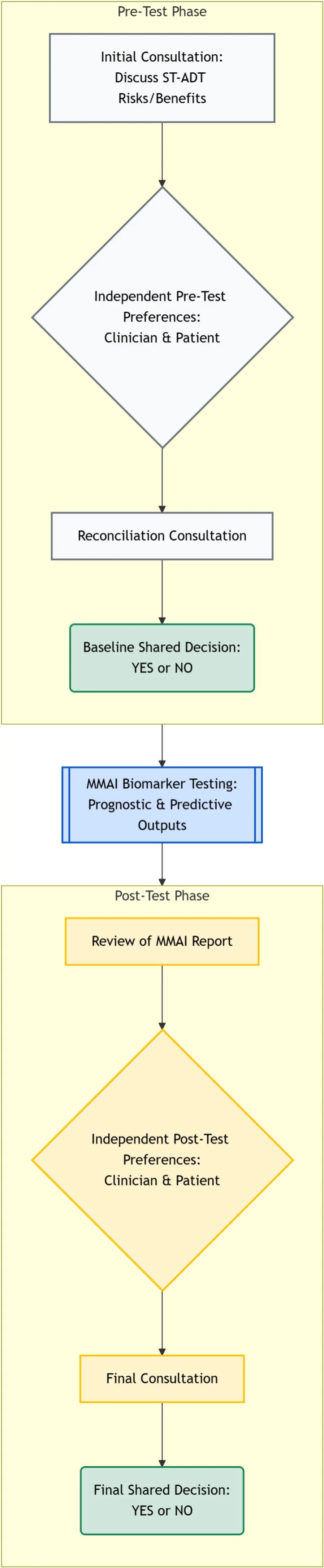
ASTuTE trial consultation workflow. The multi-step shared decision-making process for short-term androgen deprivation therapy (ST-ADT), illustrating the independent recording and subsequent reconciliation of clinician and patient preferences before (Pre-Test Phase) and after (Post-Test Phase) multimodal artificial intelligence (MMAI) biomarker testing.

The primary endpoint is the percentage of patients for whom testing led to a change in shared ST-ADT decision-making. Secondary endpoints include quality of life, cost-effectiveness, and long-term oncologic efficacy. To evaluate the primary endpoint, a structured, multi-step consultation process was utilised both before and after MMAI testing (Fig. 1). Initially, the clinician and the patient discussed the potential oncologic benefits and risks of toxicity from ST-ADT. Following this discussion, the clinician and patient independently recorded their ST-ADT preference (YES or NO). A subsequent immediate consultation was then held to reconcile these independent preferences and establish a baseline shared decision. Following MMAI testing, this sequential workflow was repeated. The MMAI test report—detailing the 10-year prognostic risk for distant metastases and a predictive indication of ST-ADT benefit (see common clinical scenarios in Supplementary Fig. 1)—was reviewed jointly. The clinician and patient again recorded their independent post-test preferences, followed by an immediate final consultation to discuss the nuances of the biomarker results and establish the final post-test shared decision.

### 2.3 Statistical analysis

The target accrual of 800 patients provides >90% power to detect a 10% change in ST-ADT utilisation. This pre-planned interim analysis evaluates the first 200 enrolled patients (December 2023 to August 2024) to assess early decisional and behavioural changes. This interim analysis assesses behavioural shifts without intent to halt the trial early, no formal stopping boundaries or alpha-spending functions were applied. The trial continues accrual to 800 patients to evaluate long-term secondary endpoints. All reported *p*-values are nominal. Differences in paired pre- and post-test ST-ADT decisions were assessed for statistical significance using McNemar's test.

## 3 Results

**Fig. 2:**
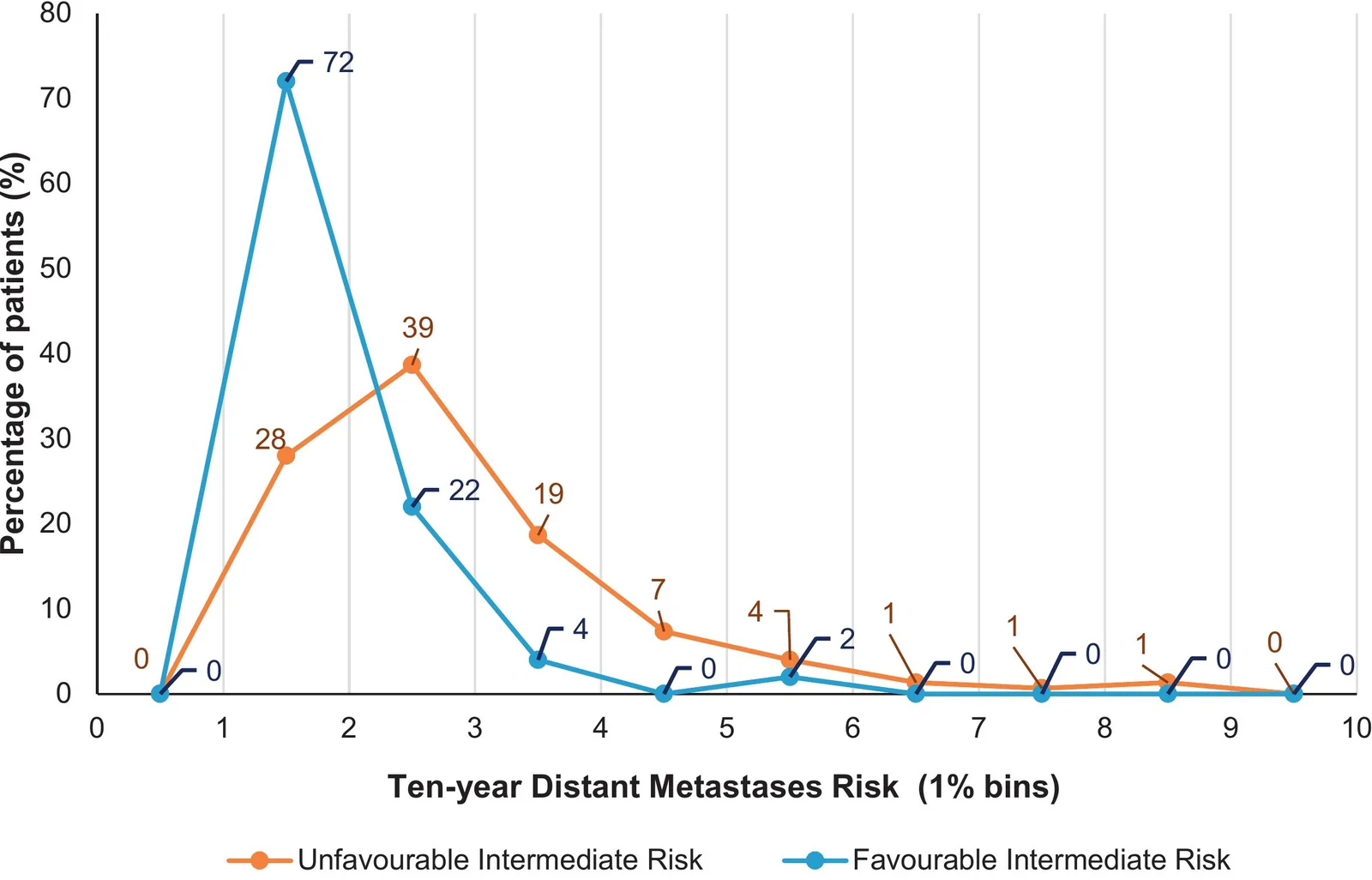
Prognostic ten-year distant metastases risks in unfavourable and favourable intermediate-risk prostate cancer patients.

Among the 200 patients, 150 were classified as National Comprehensive Cancer Network (NCCN) unfavourable intermediate-risk (UIR) and 50 as favourable intermediate-risk (FIR). Further demographics are in Table 1. The median ten-year prognostic biomarker risk for distant metastases (DM) for UIR and FIR subgroups was 2.6% (IQR 2.0–3.4) and 1.7% (IQR 1.4–2.0) respectively (*p* < 0.001; Fig. 2; with corresponding prostate cancer-specific mortality risks in Supplementary Fig. 2). There was significant overlap between risk groups, with 14/50 (28%) FIR patients that had similar risk to the median UIR for DM. Conversely, there were 42/150 (28%) UIR which had a favourable ten-year DM risk similar to the FIR median. This reflects what we see in clinical practice with heterogeneity seen among the NCCN classification.

**Table 1:** Demographics.

Parameter	FIR *n* = 50	UIR *n* = 150
Median age, yr (IQR)	71 (68–75)	74 (71–77)
T stage *n* (%)		
T1c	29 (58)	38 (25)
T2a	21 (42)	38 (25)
T2b		30 (20)
T2c		44 (30)
PSA *n* (%)		
PSA ≥ 10	2 (4)	53 (35)
PSA < 10	48 (96)	97 (65)
Total core involvement *n* (%)		
<50%	50 (100)	100 (66)
≥50%		50 (33)
ISUP *n* (%)		
ISUP 1	2 (4)	
ISUP 2	48 (96)	62 (41)
ISUP 3		88 (59)
Median 10-year PCSM risk (IQR)	0.8 (0.6–0.9)	1.2 (0.9–1.6)
Median 10-year DM risk (IQR)	1.7 (1.4–2.0)	2.6 (2.0–3.4)
Predictive Biomarker *n* (%)		
Positive	7 (14)	23 (15)
Negative	43 (86)	127 (85)

FIR = favourable intermediate-risk; UIR = unfavourable intermediate-risk; IQR = interquartile range; PSA = prostate-specific antigen; ISUP = International Society of Urological Pathology; PCSM = prostate cancer specific mortality; DM = distant metastases.

For the predictive biomarker, 30/200 (15%) suggested a potential benefit for ST-ADT, with similar rates between FIR (7/50, 14%) and UIR (23/150, 15%) groups. Conversely, the biomarker suggested no benefit for ST-ADT in 170/200 (85%). Across the entire cohort, only eight patients (8/200, 4%) fell into the higher prognostic risk group (defined as ≥80th percentile risk relative to patients within the same NCCN group). Of these, only five (5/8, 63%) were biomarker positive for ST-ADT benefit.

**Fig. 3:**
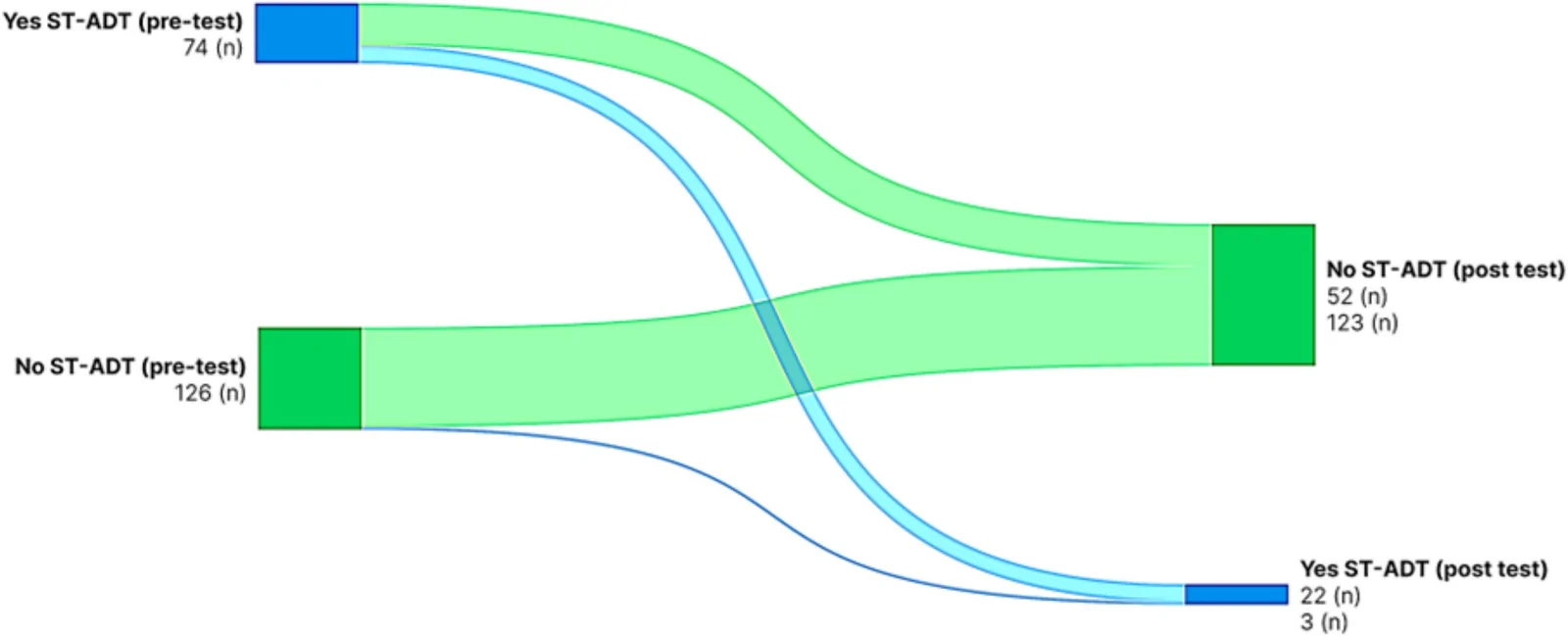
Shared short term-ADT decision choices, pre and post MMAI test results.

Across the entire cohort of 200 patients, the primary endpoint, a change in shared ST-ADT decision following MMAI testing occurred in 27.5% of cases (55/200; 95% CI: 21.4–34.2%). This interim magnitude of decision change exceeds the 10% threshold the full trial was originally powered to detect. When analysing by pre-test preference, 74 patients had an initial shared ST-ADT decision of YES. Post-test, 52 of these changed their shared decision to NO (decision change 70.3%; 95% CI: 58.5–80.3%). Conversely, 126 patients had a pre-test shared ST-ADT decision of NO. Post-test, only 3 changed their decision to YES (decision change 2.4%; 95% CI: 0.5–6.8%; Fig. 3). This difference was statistically significant (*p* < 0.001).

**Fig. 4:**
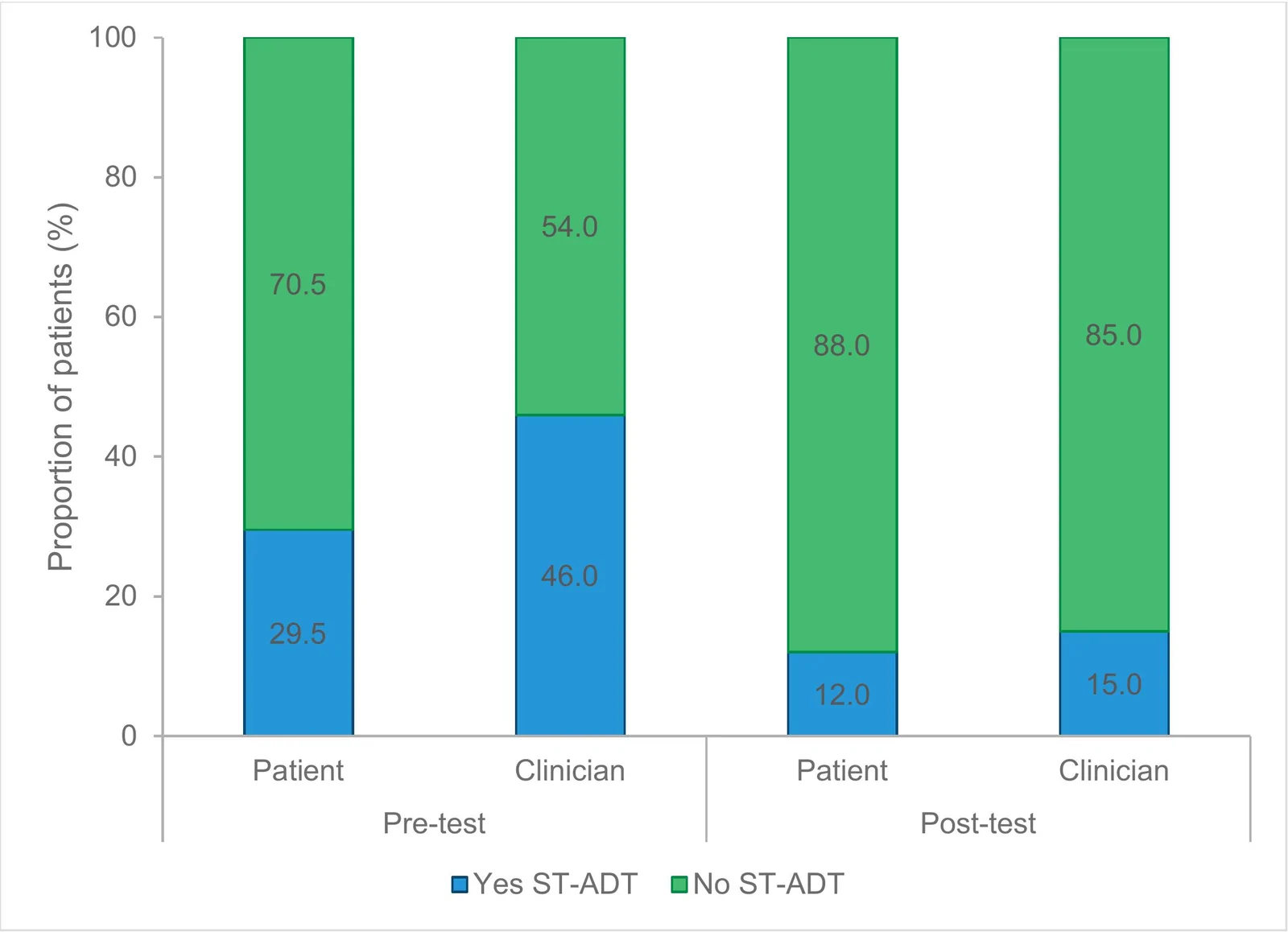
Patient and clinician short term-ADT decision choices, pre and post MMAI test results.

The initial clinician opinion for ST-ADT decision of YES was 92/200 (46%), compared to the patient opinion for ST-ADT, 59/200 (30%), implying that patients are less likely to want ST-ADT compared to clinicians (*p* < 0.001). This difference lessened post-test with clinician opinion for ST-ADT decision of YES now being 30/200, compared to patient election for ST-ADT 24/200 (15% vs 12%, *p* = 0.38; Fig. 4 and Table 2). For the predictive biomarker-positive cohort, 15/30 had an initial shared ST-ADT decision of YES and 15/30 of NO. Post-test, 13 ultimately elected for ST-ADT (12 maintained YES, 1 changed from NO to YES), while 17/30 (57%) had a final decision of NO (14 maintained NO, 3 changed from YES to NO), meaning the majority ultimately did not receive the therapy the test predicted they would benefit from. The median age was slightly older for this group, 75, compared with 73.5 for the remainder, but not significantly different (*p* = 0.45). This group also had a lower 10-year DM risk, (2.5%; IQR 1.9–2.9), compared to the 13/30 (43%) in the biomarker positive cohort who had a post-test shared ST-ADT decision of YES (5.1%; IQR 4.1–5.7). This divergence was heavily driven by the absolute prognostic score, which the MMAI categorises as Low (<3%), Intermediate (3–10%), or High (>10%) risk for 10-year distant metastasis. Among the biomarker-positive patients with a ‘Low’ prognostic risk, 12/13 (92%) ultimately decided against ST-ADT. Conversely, for those with an ‘Intermediate’ prognostic risk, 12/17 (71%) elected to receive ST-ADT. This reveals that when relative predictive benefit and absolute prognostic risk provide competing signals, clinicians and patients overwhelmingly default to the absolute prognostic risk to guide decision-making. Overall, 85.5% of final shared decisions aligned with the predictive biomarker. A greater magnitude of change was observed for UIR versus FIR patients (35%v4%; p < 0.001).

**Table 2:** Pre- and post-test ST-ADT ‘YES’ decisions by stream.

Decision stream	Pre-test ST-ADT ‘YES’ n (%)	Post-test ST-ADT ‘YES’ n (%)
Clinician	92 (46.0)	30 (15.0)
Patient	59 (29.5)	24 (12.0)
Shared Decision	74 (37.0)	25 (12.5)

## 4 Discussion

The findings from this interim analysis of an Australian multicentre implementation trial demonstrate that incorporating MMAI biomarkers significantly impacts shared decision-making regarding ST-ADT with >70% of patients who initially elected for ST-ADT ultimately deciding against it. This is a substantial reduction in the shared ST-ADT recommendation for IR-PC patients, potentially leading to significant improvements in quality of life and health savings. Additionally, the magnitude of decision change was significantly greater in the NCCN UIR group compared to the FIR group. This may reflect that UIR patients are more frequently recommended ST-ADT upfront based on guidelines, leaving more potential for de-escalation when the biomarkers indicate limited predictive benefit. This represents a significant opportunity to reduce unnecessary exposure to ADT-associated toxicities, potentially improving patient quality of life and highlights the utility of MMAI biomarkers as a de-intensification tool in this population with only 15% being predictive biomarker positive. The FIR/UIR split, although prognostic, did not predict for ST-ADT benefit. The lower absolute benefit for FIR men was incorporated into shared decision-making with still high concordance (85.5%) between the final shared ST-ADT decision and the predictive biomarker status. By reducing clinician-patient discordance on ST-ADT, these biomarker tests can improve shared decision-making. The objective, personalised report can help bridge differing views, boosting confidence in the treatment decision and strengthening the clinician-patient relationship. The majority of this cohort was classified as NCCN UIR, a population for whom guidelines typically recommend ST-ADT, the biomarker-derived absolute DM risk remained low (median 10-year DM risk 1.7% to 2.6%). This low baseline absolute risk inherently predisposed the cohort to de-escalation, which must be considered when generalising the magnitude of this effect. While the NCCN intermediate-risk classification is prognostically relevant, our data, consistent with previous literature [6] and clinical experience, underscore its inherent heterogeneity of this population and the need for better tools.

This study's strengths include its multicentre, Australian-wide implementation trial design, which provides real-world data on a large number of varied radiation oncologists' practices. This trial continues accrual toward 800 patients with further secondary endpoints of quality of life, cost effectiveness, and efficacy. This trial allows for a wide range of radiotherapy schedules [7], including SBRT boosts [8] and focal boosts [9] to enable data generalisability across these modern techniques. The predictive MMAI model was derived from historical NRG/RTOG cohorts treated primarily with conventional-dose radiotherapy. With modern dose-escalated schedules potentially attenuating the incremental benefit of ADT, evaluating the tool's performance within contemporary cohorts is a core strength of this trial's prospective design. As long-term oncological outcomes mature, this cohort will provide critical data to confirm the predictive biomarker's continued utility and generalisability within the context of modern, dose-escalated radiotherapy practices. The clinical utility of MMAI biomarkers extend beyond its application in standard practice and is now being integrated into ongoing clinical trial protocols, such as the NINJA trial, to further refine treatment selection and ADT necessity [10].

### 4.1 Limitations

As an interim analysis of an implementation trial, this study evaluates behavioural and decisional shifts rather than definitive clinical endpoints. Although mature oncologic outcomes have yet to be reported for this cohort, the clinical validity of the MMAI is supported through post-hoc RTOG 9408 analysis and its inclusion as a risk stratification option within the NCCN guidelines [4], [11]. Consequently, the ultimate goal of reporting future long-term outcomes from this trial is to translate this established historical validation into prospective, real-world clinical utility. The MMAI tool is currently being assessed in a prospective investigational context rather than as an established standard of care in Australia. The open-label, single-arm nature of the registry introduces potential bias. Without a control group, it is possible clinicians were influenced by the novelty of the trial setting (the Hawthorne effect) and more inclined to withhold ADT when supported by the AI. The majority of test results indicated ‘less benefit’ in 85% of cases, and there was a consistent directional pull toward de-escalation. When combined with the open-label setting and the implementing network's collaborative research partnership with the test vendor, these non-causal explanations should be considered. Consequently, while the substantial magnitude of the observed decision change strongly indicates a real-world clinical impact, these potential biases limit the ability to interpret the behavioural shift. Finally, our current data lacks comprehensive capture of patient comorbidities, baseline functional status, and varying levels of health literacy during the consultation process. ADT omission is heavily influenced by a patient's pre-existing health profile, and the absence of this information restricts our ability to isolate how much of the decision change was driven independently by the biomarker versus baseline health constraints. Despite this, we believe the quality-of-life data will still be able to highlight this difference. While this interim analysis focuses on decisional impact, we recognise that patient-reported quality-of-life metrics and health economic analyses are critical for establishing true clinical utility and driving widespread adoption. These remain pre-specified secondary endpoints of the ASTuTE trial, and comprehensive cost-effectiveness and mature PROs will be reported in future analyses as the dataset matures.

## 5 Conclusion

These interim results provide real-world evidence that MMAI biomarkers significantly influence shared ST-ADT decision-making for IR-PC, leading to a substantial reduction in ADT use, particularly among patients and clinicians initially inclined toward it. This further supports the biomarker tests' clinical utility in refining patient selection within this heterogeneous population. Continued accrual and longer-term follow-up are essential to confirm these findings and evaluate the impact of this test on oncologic outcomes.

## CRediT authorship contribution statement

**Eric Wegener:** Writing – review & editing, Writing – original draft, Visualization, Validation, Project administration, Methodology, Investigation, Formal analysis, Conceptualization. **Michael Ng:** Writing – review & editing, Investigation, Funding acquisition. **Mario Guerrieri:** Writing – review & editing, Investigation, Funding acquisition. **Timothy N. Showalter:** Resources, Project administration, Funding acquisition, Conceptualization. **Jeremy de Leon:** Writing – review & editing, Investigation, Funding acquisition. **Sagar Ramani:** Writing – review & editing, Investigation, Funding acquisition. **Marcus Dreosti:** Writing – review & editing, Investigation, Funding acquisition. **Tee Lim:** Writing – review & editing, Investigation, Funding acquisition. **Bradley Wong:** Writing – review & editing, Investigation, Funding acquisition. **Annie Ho:** Writing – review & editing, Investigation, Funding acquisition. **Michael Chao:** Writing – review & editing, Investigation, Funding acquisition. **Zachary Wilczynski:** Resources, Project administration, Funding acquisition. **Peter Chong:** Writing – review & editing, Investigation. **Scott McClintock:** Writing – review & editing, Investigation. **Darren Foreman:** Writing – review & editing, Investigation. **Matthew Brown:** Writing – review & editing, Investigation. **Stephen McCombie:** Writing – review & editing, Investigation. **Kevin McMillan:** Writing – review & editing, Investigation. **Marcus Handmer:** Writing – review & editing, Investigation. **Mark Frydenberg:** Writing – review & editing, Investigation. **Lih-Ming Wong:** Writing – review & editing, Investigation. **Dickon Hayne:** Writing – review & editing, Investigation. **John Yaxley:** Writing – review & editing, Investigation. **Phillip Stricker:** Writing – review & editing, Investigation. **Jarad Martin:** Writing – review & editing, Writing – original draft, Validation, Supervision, Methodology, Investigation, Formal analysis, Conceptualization.

## Declaration of competing interest

The authors declare the following financial interests/personal relationships which may be considered as potential competing interests: TS is Chief Medical Officer and ZW is Director/Product Management at ArteraAI®. The authors: EW, MN, MG, JdL, SR, MD, TL, BW, AH, MC, and JM are employed by GenesisCare. The ASTuTE trial was financially supported by GenesisCare and conducted in a collaborative research partnership with ArteraAI® to facilitate the biomarker testing.

## Appendix A Supplementary data

Supplementary material Multimodal Artificial Intelligence (MMAI) Test Report Scenarios and Prostate Cancer-Specific Mortality Risk Distributions.
