# Supplementary material for: Artificial Intelligence impact on decision-making regarding short-term ADT with prostate radiotherapy: ASTuTE clinical trial interim results
Source: Clin Transl Radiat Oncol. 2026 Aug 10;61:101257. doi: 10.1016/j.ctro.2026.101257 (PMC13520630; doi:10.1016/j.ctro.2026.101257)
Supplement: Supplementary file 1 [file mmc1.docx]

**SUPPLEMENTARY**

**Supplementary Fig. 1:** MMAI Test report examples in four common clinical scenarios. The green box highlights the 10-year risk of distant metastasis, reported as a continuous variable with low, intermediate, and high-risk categories. The blue box highlights the 5-year risk of distant metastasis and 10-year risk of prostate cancer-specific mortality to help inform decision-making; separate prognostic models are used to calculate these risks. The purple box highlights the predictive biomarker test result for ADT benefit.

**A) Scenario A – initial PSA 9.72, ISUP 3, T1c. Prognostic for a Low 2% 10-year risk of distant metastasis with short-term ADT to be of ‘less benefit’. Anticipated very low absolute benefit with the addition of ADT.**

**B)** **Scenario B – initial PSA 12.8, ISUP 2, T2c. Prognostic for a High 12% 10-year risk of distant metastasis with short-term ADT to be of ‘more benefit’. Anticipated very high absolute benefit with the addition of ADT.**

**C) Scenario C – initial PSA 11.42, ISUP 3, T2a. Prognostic for a Low 2% 10-year risk of distant metastasis with short-term ADT to be of ‘more benefit’. Anticipated low absolute benefit with the addition of ADT.**

**D) Scenario D – initial PSA 6.46, ISUP 2, T2a. Prognostic for a High 11% 10-year risk of distant metastasis with short-term ADT to be of ‘less benefit’. Anticipated low absolute benefit with the addition of ADT.**

**Supplementary Fig. 2: Prostate cancer-specific mortality ten-year risk in unfavourable and favourable intermediate-risk prostate cancer patients**

Percentage of patients (%)

| Prostate Cancer-Specific Mortality Ten-year Risk (1% bins) | Unfavourable Intermediate Risk | Favourable Intermediate Risk |
| --- | --- | --- |
| 0.25 | 0.666667 | 2 |
| 0.75 | 34 | 78 |
| 1.25 | 35.3333 | 14 |
| 1.75 | 18.6667 | 4 |
| 2.25 | 5.33333 | 0 |
| 2.75 | 2.66667 | 2 |
| 3.25 | 1.33333 | 0 |
| 3.75 | 0.666667 | 0 |
| 4.25 | 1.33333 | 0 |
| 4.75 | 0 | 0 |

The prostate cancer-specific mortality ten-year risk was similar to the ten-year distant metastases risk. The median ten-year prostate cancer-specific mortality was 1.2% (IQR 0.9 – 1.6) and 0.8% (IQR 0.6 – 0.9) in the UIR and FIR subgroups, respectively, which was significantly different (p<0.001).
